# Unravelling driver genes as potential therapeutic targets in ovarian cancer via integrated bioinformatics approach

**DOI:** 10.1186/s13048-024-01402-7

**Published:** 2024-04-23

**Authors:** Anam Beg, Rafat Parveen, Hassan Fouad, M. E. Yahia, Azza S. Hassanein

**Affiliations:** 1 Department of Computer Science, Jamia Millia Islamia, New Delhi, 110025 India; 2 Applied Medical Science Department, CC, King Saud University, Riyadh, 11433 Saudi Arabia; 3 Abu Dhabi Polytechnic, Institute of Applied Technology, Abu Dhabi, 111499 United Arab Emirates; 4 Biomedical Engineering Department, Faculty of Engineering, Helwan University, Cairo, Egypt

**Keywords:** Ovarian cancer, Hub genes, Prognostic relevance, RNA-binding proteins, Docking, MD simulation therapeutic targets

## Abstract

Target-driven cancer therapy is a notable advancement in precision oncology that has been accompanied by substantial medical accomplishments. Ovarian cancer is a highly frequent neoplasm in women and exhibits significant genomic and clinical heterogeneity. In a previous publication, we presented an extensive bioinformatics study aimed at identifying specific biomarkers associated with ovarian cancer. The findings of the network analysis indicate the presence of a cluster of nine dysregulated hub genes that exhibited significance in the underlying biological processes and contributed to the initiation of ovarian cancer. Here in this research article, we are proceeding our previous research by taking all hub genes into consideration for further analysis. GEPIA2 was used to identify patterns in the expression of critical genes. The KM plotter analysis indicated that the out of all genes 5 genes are statistically significant. The cBioPortal platform was further used to investigate the frequency of genetic mutations across the board and how they affected the survival of the patients. Maximum mutation was reported by ELAVL2. In order to discover viable therapeutic candidates after competitive inhibition of ELAVL2 with small molecular drug complex, high throughput screening and docking studies were used. Five compounds were identified. Overall, our results suggest that the ELAV-like protein 2-ZINC03830554 complex was relatively stable during the molecular dynamic simulation. The five compounds that have been found can also be further examined as potential therapeutic possibilities. The combined findings suggest that ELAVL2, together with their genetic changes, can be investigated in therapeutic interventions for precision oncology, leveraging early diagnostics and target-driven therapy.

**Supplementary Information:**

The online version contains supplementary material available at 10.1186/s13048-024-01402-7.

## Introduction

Ovarian cancer is presently ranked as the fifth most prominent cause of cancer-related mortality in women within the United States [1]. Globally, an estimated 140,000 women succumb to ovarian cancer annually. In most countries, the rate of survival over a five-year period of OC is less than 40% [2]. According to GLOBOCAN 2020 datasheets, INDIA ranks third in terms of the number of incidence and death cases [3]. The manifestation of this incapacitating illness is characterized by subtle symptoms, and upon diagnosis, the available therapeutic interventions frequently exhibit constraints. In order to optimize patient care, healthcare practitioners must possess fundamental knowledge regarding the manifestations and indicators of ovarian cancer, as well as the array of diagnostic imaging modalities and therapeutic interventions at their disposal [4]. Ovarian cancer lacks a screening test, resulting in a tendency for late-stage diagnosis and subsequently elevated rates of recurrence within certain demographic area [5]. The process of early diagnosis encompasses a spectrum of approaches, ranging from recognizing the general symptoms commonly linked with cancer to undertaking preventative surgical interventions for the removal of potentially vulnerable tissue [5]. Despite ongoing progress, ovarian cancer continues to be the most lethal of female gynecologic malignancies. Over 70% of OC patients are detected at a stage that is advanced because of the ambiguous symptoms [6]. More than 21,400 patients received ovarian cancer diagnosis in 2021. Nine out of ten of these instances are epithelial ovarian cancer. A crucial factor throughout the poor prognosis and increased mortality is the lack of early and efficient diagnosis procedures [7, 8]. It is therefore creating more efficient diagnosing and treatment strategies and more efforts must be made to find and better understand novel biomarkers and specific OC targets [9, 10].

In recent times, the utilization of gene profiling and gene arrays has been employed for the purpose of identifying genes that exhibit different expression patterns [11–13]. In the present era, the widespread adoption of high-throughput microarray technology and the use of bioinformatics analysis have become increasingly common in the detection of gene expression differences between malignant and non-cancerous tissues. Additionally, this methodology aids in the evaluation of genes that are differentially expressed (DEGs) and the clarification of the pathways involved in the process of carcinogenesis and the development of cancer [13, 14]. The investigation of gene expression profiles associated with various types of cancer has led to the identification of novel biomarkers and therapeutic targets [15–17]. These findings have demonstrated consistent efficacy in clinical studies. Re-examining these data could lead to the discovery of novel viewpoints on ongoing OC research [18]. The limitations of existing biomarker studies and the potential inconsistencies in differential expression gene (DEG) results may be attributed to the diverse variety of tumors and the intricate molecular regulatory mechanisms of ovarian cancer (OC). In addition, a significant proportion of treatment interventions that are unsuccessful, together with the limited overall survival (OS) and progression-free survival (PFS) observed in patients with ovarian cancer (OC), can be attributed to the development of drug resistance, which continues to provide a substantial challenge [19]. Due to the previously demonstrated efficacy and reliability of multiple bioinformatics investigations pertaining to ovarian cancer (OC), the utilization of bioinformatics analysis holds potential in facilitating the exploration of biomarkers and molecular targets implicated in the onset and advancement of disease [18, 20, 21].

In this study, we are extending our prior research findings [11] by validating hub genes through the utilization of various computational techniques. The expression of approved genes was subjected to survival and mutational analysis using the KM plotter, GEPIA2 and cBioPortal. The genes that were chosen were subjected to additional investigation for the purpose of mutation identification. The genes exhibiting the greatest number of mutations were selected for further research. Subsequently, specific treatment interventions were discovered for ovarian cancer targeting certain genes. The molecular drug targets will be subjected to validation by molecular dynamic simulation.

## Materials and methods

### Data sources of hub genes and statistical analysis

The microarray dataset GSE119055 was utilized in a prior study and obtained from the public Gene Expression Omnibus (GEO) database of the National Center for Biotechnology Information (NCBI) for the purpose of conducting differential expression analysis. The dataset can be accessed at the following link: https://www.ncbi.nlm.nih.gov/geo/query/acc.cgi?acc=GSE119055. The transcriptome data underwent transformation and normalization using R language version 3.5.0 (https://www.r-project.org/), in conjunction with the resources provided by Bioconductor (http://www.bioconductor.org/). The miRNA expression data underwent pre-processing using the “RMA” methodology, which involved background adjustment and normalization utilizing the quantile method. The selection of specific digital elevation models (DEMs) was performed using a T static technique, employing the Linear Models for Microarray (limma) package from Bioconductor. In addition, the annotation of DEMs was performed utilizing the output package, which encompassed an annotation table. The p values were subjected to the Benjamini-Hochberg (BH) false discovery rate methods for analysis. The differentially expressed miRNAs (DEMs) were identified based on the criteria of |logFC (fold change)| > 2 for upregulated miRNAs and ≤ − 2 for downregulated miRNAs, as well as a p-value < 0.05 as the primary cut-off. We identified differentially expressed genes by utilizing significant p-values and log fold changes.A total of 9 hub genes were selected (MAF, ZNF532, CADM1, SCN2A, BCL2, ELAVL2, PRKACB and TAOK1 and ESRRG), from previous published research article [11]. Here in this research article, we will proceed our previous research article via validation through GEPIA2, survival analysis through Km plotter and mutational analysis through cBioportal. The significant genes will be selected for further analysis.

### Validation of driver genes

The website GEPIA (http://gepia.cancer-pku.cn/detail.php) is a valuable resource for researchers [22]. GEPIA2 represents an enhanced iteration of GEPIA, designed to facilitate the analysis of RNA sequencing expression data derived from a substantial cohort of 9,736 tumor samples and 8,587 normal samples sourced from the TCGA (http://portal.gdc.cancer.gov/) and the GTEx (http://gtexportal.org/home/) databases [23]. These samples originate from the TCGA and GTEx projects and are subjected to a standardized processing pathway for consistent and reliable results. GEPIA2 offers a range of customisable features, including the examination of differential expression between tumor and normal samples, the ability to profile data based on cancer kinds or pathological stages, patient survival analysis, identification of similar genes, correlation analysis, and dimensionality reduction analysis.

The validation of hub genes was conducted using the web-based tool GEPIA 2. This involved comparing the relative expression of these genes in normal tissue samples and ovarian cancer tissue samples obtained from The Cancer Genome Atlas (TCGA) and Genotype-Tissue Expression (GTEx) databases, respectively. The following parameters were utilized for boxplot comparison. p-Value cutoff was 0.05, jittersize was kept at 0.4, logFc ≥ 2 for up- and **≤ −** 2 for downregulated genes [11].

### Survival analysis of hub gene

The Kaplan-Meier plotter (also known as the KM plotter) is a graphical portrayal of survival analysis that is used in medical research. The KM estimates of survival probabilities at time t and is calculated as follows [24]:

$$\,{\rm{S}}\left( {\rm{t}} \right){\rm{ = \Pi }}\left[ {{\rm{i = 1 to j}}} \right]{\rm{ }}\left( {{\rm{1 -- }}{{\rm{d}}_{\rm{i}}}{\rm{/ }}{{\rm{n}}_{\rm{i}}}} \right)$$

where:

S(t) represents the expected survival probability at t time.

j is count of events (such as mortality) that have occurred by t time.

d_i_ is count of events that occurred at t_i_ time.

n_i_ represents count of individuals at risk just before to t_i_ time (i.e., those who have not yet experienced an event or been censored).

The formula can be represented graphically with a step function, with the predicted survivor probability plotted on the y-axis and time plotted on the x-axis. The resulting graphic can be used to compare survival rates between groups, such as individuals who received different therapies.

The KM plotter can evaluate the predictive value of genes on survival for a variety of different malignancies, (http://kmplot.com/analysis/). Patients with OC were split into two groups on the basis of the expression of a certain gene: high-expression and low-expression The prognostic implications of ovarian cancer-associated hub genes were investigated using the KM plotter database [25]. This database contains information on expression of genes, overall survival of patients and relapses for 21 distinct types of cancer from databases like TCGA, GEO and EGA. The samples were separated into two different levels of expression groups based on the median projected values of each hub gene in order to examine the OS of OC patients [26]. Affymetrix IDs were assigned to each hub gene, and after deleting outlier arrays, the KM survival graphs corresponding to those IDs were produced. Moreover, information on the graph included the log-rank p values, hazard ratio (HR), number-at-risk, and 95% confidence interval (95% CI).

The survival function, denoted as S(t), is a mathematical representation that quantifies the chance of an individual or a system surviving until a given time point, t. The hazard function, denoted as h(t), quantifies the conditional probability of mortality at a given time t, given that an individual has survived up to that point. The graph depicting the relationship between the survival function S(t) and time t is commonly referred to as the survival curve. The estimation of this curve can be derived from observed survival times via the Kaplan-Meier method, which obviates the need for assuming an inherent probability distribution. The methodology is based on the fundamental principle that the likelihood of survival for k or more time intervals subsequent to the initiation of the study can be determined by multiplying the k observed survival rates for each time period (i.e., the mean number of individuals that survive), as articulated by:

$${\rm{S}}\left( {\rm{k}} \right){\rm{ = }}{{\rm{p}}_{\rm{1}}}{\rm{ \times }}{{\rm{p}}_{\rm{2}}}{\rm{ \times }}{{\rm{p}}_{\rm{3}}}{\rm{ \times \ldots \times }}{{\rm{p}}_{\rm{k}}}$$

In this context, let p_1_ represent the proportion of individuals who successfully endure the initial period, whereas p_2_ denotes the proportion of individuals who continue to survive subsequent periods, given that they have survived the first period. This pattern continues for subsequent periods. The proportion of individuals who have survived from period i to period i is given by:

$${{\rm{p}}_{\rm{i}}}{\rm{ = }}{{\rm{r}}_{\rm{i}}}{\rm{ - }}{{\rm{d}}_{\rm{i}}}{\rm{/}}{{\rm{r}}_{\rm{i}}}$$

Let ri represent the initial population size at a given moment, and di represent the number of individuals who have deceased within the specified period.

The hazard is the likelihood of dying based on the number of patients who have lived up to that point in time, or the danger of death at that moment in time. However, if the risk of dying in one of the groups is, for instance, twice that of another group at a given moment in time, it is predicted that the risk of dying in the other group will continue to be twice that at any later point in time. In a nutshell, the hazard ratio is not time dependent. It is difficult to explain hazard from sample data since it estimates the instantaneous danger of mortality. Instead, consider the cumulative hazard function H(t). This can be calculated using the cumulative survival function S(t).:

$${\rm{H}}\left( {\rm{t}} \right){\rm{ = -ln S}}\left( {\rm{t}} \right)$$

The genes were statistically significant if their log rank *p* < 0.05. The cBio Portal was used to study the mutational profiles of the genes that are statistically significant and having information about genetic modification.

### Genetic alteration analysis using cBioPortal

We obtained TCGA datasets of ovarian serous cystadenocarcinoma via cBioPortal (https://www.cbioportal.org). Further we explored the selected study option and entered a query to search for all the hub genes for genetic alteration features. The “Cancer Types Summary” module displayed the results of the kind of mutation, alterations frequency, and copy number alteration (CNA) across all tumors of TCGA. The 2D structures of the discovered proteins were used to map each mutation, and their frequencies were recorded. To see each mutation closely, the domain organization of the proteins’ structures was created.

### Receptor ligand preparation and molecular docking

A protein called ELAV-like protein 2 with the mass (Da) of 39,504 and an Alpha Fold structure of 359 amino acids was found in UniProt with the accession number Q12926 (https://www.uniprot.org/uniprotkb/Q12926/entry). The structure was prepared for blindly molecular docking by applying default parameter using auto dock tools resulting converted into PDBQT from having covered protein structure in XYZ dimensions. For small molecules collection ZINC database was accessed and library of several thousand molecules was used in the same extension file format PDBQT. Finally molecular docking was performed with the help of Auto Dock vina [27]. To analyse the result of molecular docking other software such as PyMOL [28] and Discovery Studio Visualizer [29] was employed to know binding pattern, interconnections between protein and ligand, involving residues and their mapping.

### Molecular dynamic simulations

The advanced technologies of molecular dynamics simulation are utilized to examine the behavior of intricate biomolecular systems. We simulated unligand protein and protein ligand complex in our work and aimed to gain insights into the structural and dynamic changes that occur within the system over a span of a 100 ns simulation [30]. At a temperature of 300 K, the simulations were run using Gromacs 2020.6 and the GROMOS96 43a1 force field. The PRODRG web server was used to produce the topology for the ligand, which is designated as ZINC03830554. With a minimal spacing of 1.0 nm between the protein and the box edge, the system was solvated in a cubic box of SPC216 molecules of water. Appropriate counter ions were added to the system to neutralize the net charge. Energy was minimized for native protein and protein bound ligand complex using steepest descent algorithm. After energy minimization the simulation protocol consisted of two main stages: (i) equilibration, and (ii) production. The equilibration stage was performed in two ensembles, NVT and NPT, for 100 ps each. During the NVT equilibration, the temperature was maintained at 300 K using the V-rescale thermostat. The NPT equilibration was performed using the Parrinello-Rahman barostat, with a reference pressure of 1 bar. A time step of 2 fs was used during both the equilibration stages. After equilibration, the system was subjected to a 100 ns production run using the same simulation parameters. The trajectory was saved every 10 ps for subsequent analysis. At a cut-off distance of 1.2 nm, the Particle Mesh Ewald (PME) approach was used to handle the long-range electrostatic interactions. The bond lengths were limited by the LINCS algorithm, which allowed for a 2-fs time step. After completion of simulations the trajectories was analyzed.

## Results

### Gene expression profiling of key DEGs

**Fig. 1:**
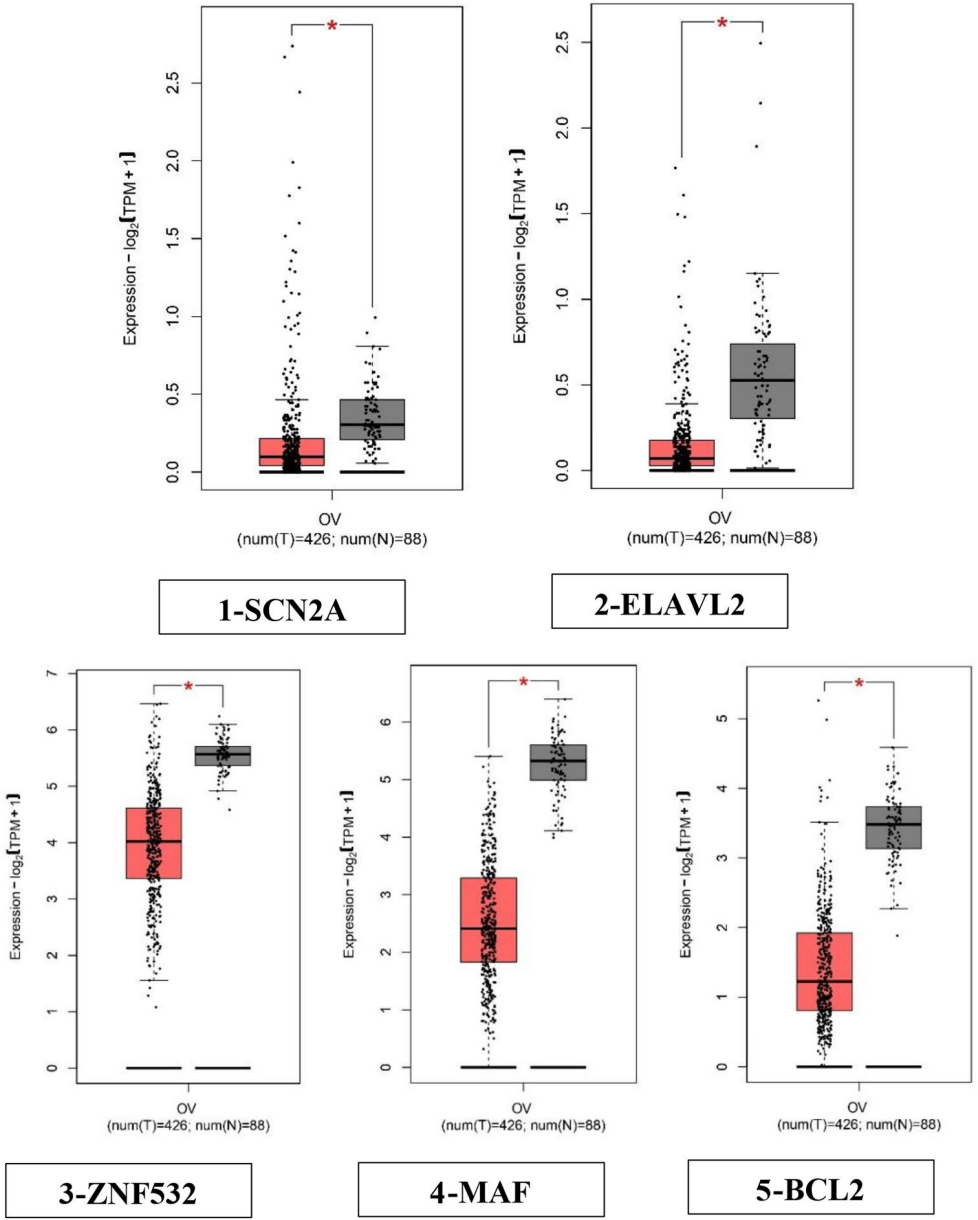
Comparisons of the expression of the five genes between ovarian cancer and normal ovarian tissues in TCGA and GTEx based on GEPIA. The Y axis represents the log2 (TPM + 1) for gene expression. The gray bar represents the normal tissues, whereas the red bar represents the tissues affected by ovarian cancer. The median is shown by a broad horizontal line positioned in the centre, while the lower and upper boundaries of each box correspond to the first and third quartiles, respectively. The lower and upper error bars correspond to the minimum and maximum values of the expression data, respectively. The red and grey boxes in the diagram correspond to ovarian cancers and normal tissues, respectively. The differential analysis was conducted using the one-way analysis of variance (ANOVA) method, where disease stage was considered as the variable for determining differential expression. Statistical significance was indicated by an asterisk, and each dot represented a unique tumor or normal sample. The values were obtained from the GEPIA database. Transcripts per kilobase million (TPM) is a metric used to quantify gene expression levels in transcriptomic studies. The box plots (1–5) depicting the expression levels of the five hub genes indicate significant dysregulation in ovarian cancer as compared to normal ovarian tissue. (**1**) SCN2A—down-regulated, (**2**) ELAVL2—down-regulated, (**3**) ZNF532—down-regulated, (**4**) MAF—downregulated (**5**), BCL2—down-regulated. All the genes were significant with *p* < 0.05

GEPIA, also known as Gene Expression Profiling Interactive Analysis, is a web-based platform that facilitates the analysis of gene expression data. The GEPIA2 platform was selected for the analysis of hub genes in ovarian cancer. This analysis focused on validating the total expression levels of these genes in normal tissues and examining their expression patterns at different stages of ovarian cancer [31]. The box plot depicted in Fig. 1 illustrates the aberrant expression of the 5 hub genes in ovarian cancer in comparison to normal ovarian tissue. The previous study observed a downregulation of the genes SCN2A, ELAVL2, ZNF532, MAF and BCL2 which were identified in ovarian cancer [11]. These 5 hub genes were validated by GEPIA2 for their expression and further analysed via KmPlotter for survival analysis.

### Survival analysis data

**Fig. 2:**
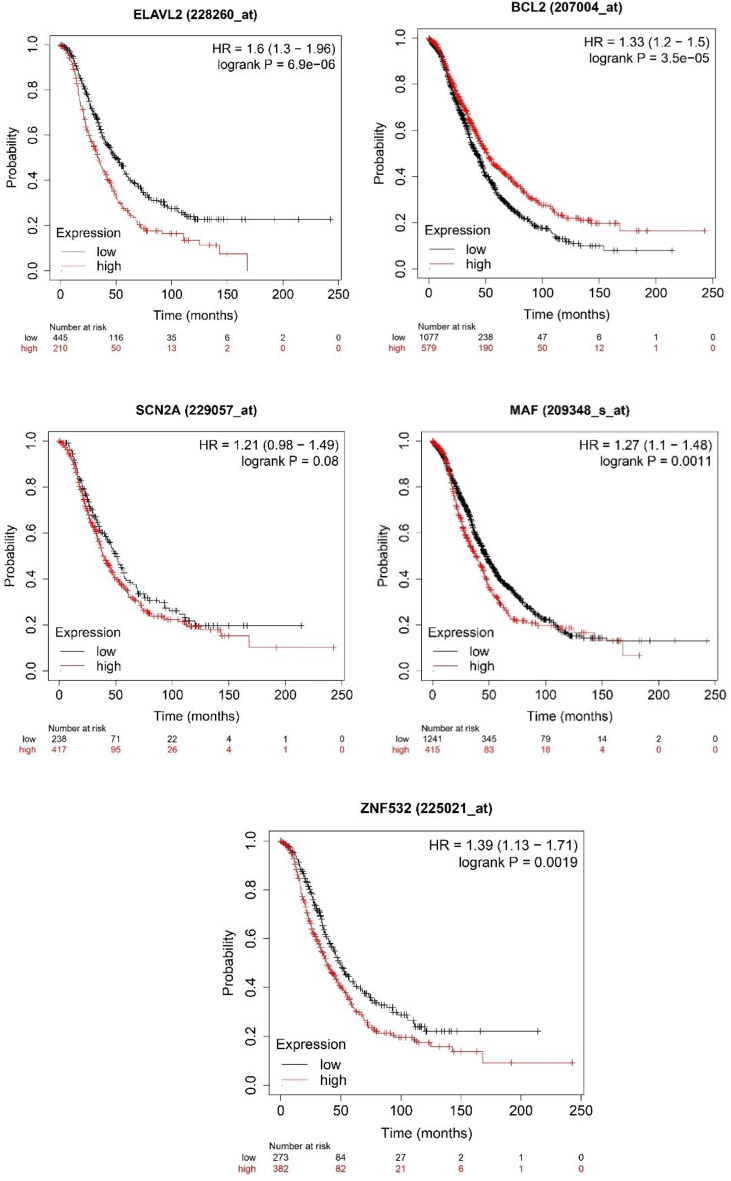
Analysis of the prognostic value of all the different five genes using KM-Plotter Plots shown in Figure displays the prognostic relevance of all the 5 hub genes

The prognostic information of our five hub genes were determined using the KM plotter (SCN2A, ELAVL2, BCL2, MAF, and ZNF532,) to confirm the association between patterns of expression and metastasis risk in ovarian cancer patients. In this analysis we have selected genes that are significant. The KM plots shown in Fig. 2(A–I) depicted the low expression levels of all the 5 driver genes. **SCN2A** (HR = 1.21; 95% CI = 0.98–1.49; *p* > 0.05, **ELAVL2** (HR = 1.6; 95% CI = 1.3–1.96; *p* < 0.05), **ZNF532** (HR = 1.39; 95% CI = 1.13–1.71; *p* < 0.05), **MAF** (HR = 1.27; 95% CI = 1.1–1.48; *p* < 0.05), **BCL2** (HR = 1.33; 95% CI = 1.2–1.5; *p* < 0.05). The p values show the significance of genes that is being taken into account. Table 1 displays the p value of each hub gene with significant genes taken into discussion in this research article.

In the context of a Kaplan-Meier (KM) plot or survival analysis, the Hazard Ratio (HR) the result in case of ELAVL2 is interpreted as for e.g.- “1.6 (1.3–1.96),” indicates the relative risk of an event occurring in one group compared to another group.

Point Estimate (HR = 1.6): The point estimate of the Hazard Ratio is 1.6. This means that the group associated with this HR value has a 1.6 times higher risk of experiencing the event of interest (e.g., death, disease recurrence) compared to the reference group. In other words, the event is 60% more likely to occur in the first group than in the reference group.

Confidence Interval (1.3–1.96): The confidence interval provides a range of values within which we can reasonably expect the true Hazard Ratio to fall with a certain level of confidence. In this case, the 95% confidence interval for the Hazard Ratio is between 1.3 and 1.96. This means that we are 95% confident that the true Hazard Ratio lies within this range. If the confidence interval does not include the value 1 (which indicates no difference in risk), it suggests that the observed difference is statistically significant.

If we interpret the result we may conclude that the point estimate (HR = 1.6) indicates that the group being compared has a 1.6 times higher risk of the event compared to the reference group.The lower limit of the confidence interval (1.3) suggests that the risk is at least 1.3 times higher, while the upper limit (1.96) suggests that the risk could be as much as 1.96 times higher.

In simpler terms, the data suggests that the group with the higher risk (as indicated by the Hazard Ratio) is statistically significantly more likely to experience the event of interest compared to the reference group. The magnitude of the risk increase falls within the range of 30% (1.3) to 96% (1.96) higher risk, based on the confidence interval.

Among the five hub genes examined, four genes were shown to be statistically significant. Subsequently, the four genes underwent further examination to identify any alterations. The cBio Cancer Genomics Portal was used to further investigate the highest mutation summary. The gene with the greatest number of mutations will be selected for subsequent study.

**Table 1:** All the significant genes are taken into consideration for cBio portal analysis

S.No	Genes	Log rank P	Significance
1	SCN2A	0.08	No
2	ELAVL2	6.6e-0.6	Yes
3	ZNF532	0.0019	Yes
4	MAF	0.0011	Yes
5	BCL2	3.5-05	Yes

### **cBioPortal analysis of hub DEGs**-**Genetic alteration data analysis**

**Fig. 3:**
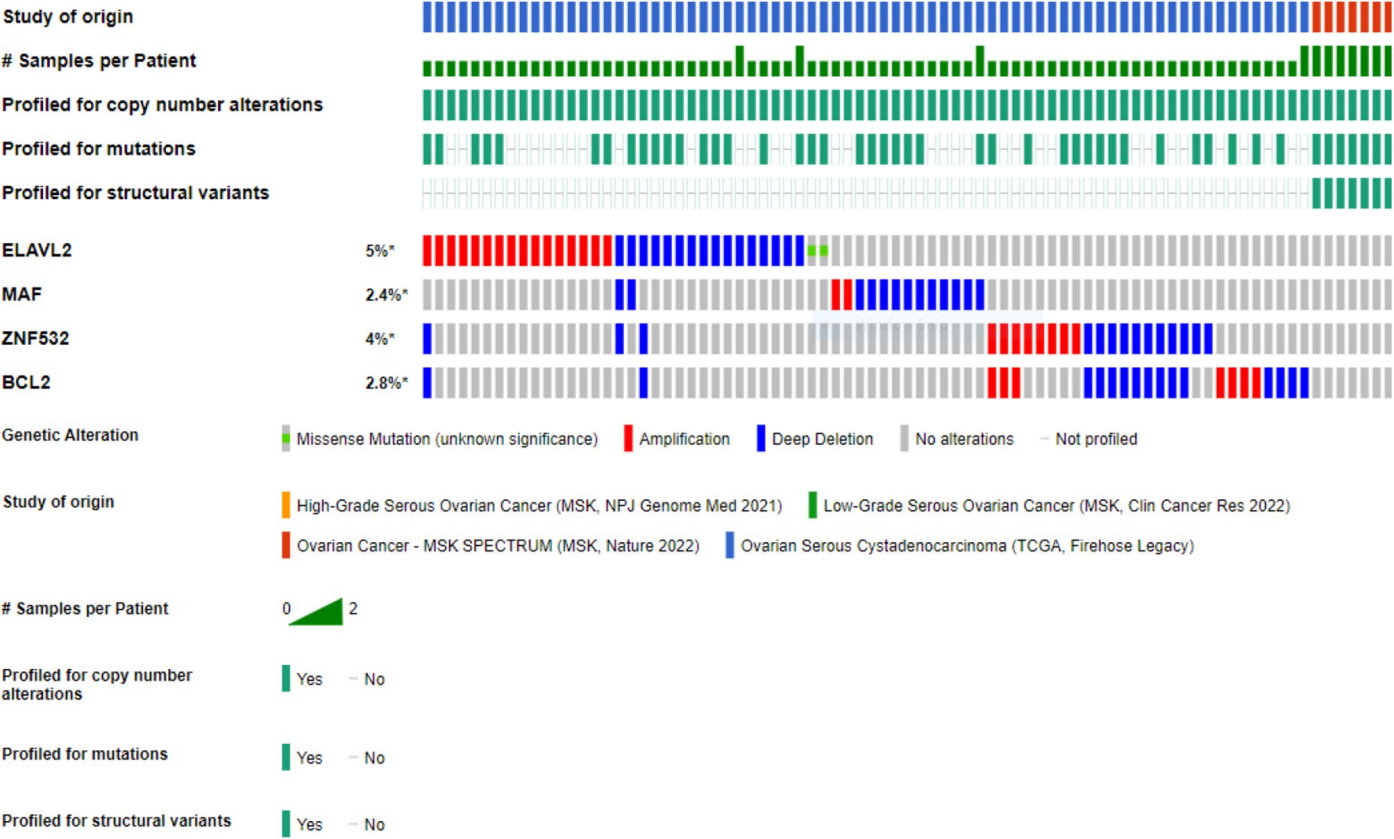
OncoPrint highlights genomic alterations among samples. The rows are the hub DEGs, while the vertical columns are tumor samples. Red, blue, green, yellow, and gray colored bars represent amplification, deep deletion, missense, splice, and truncating mutations

**Fig. 4:**
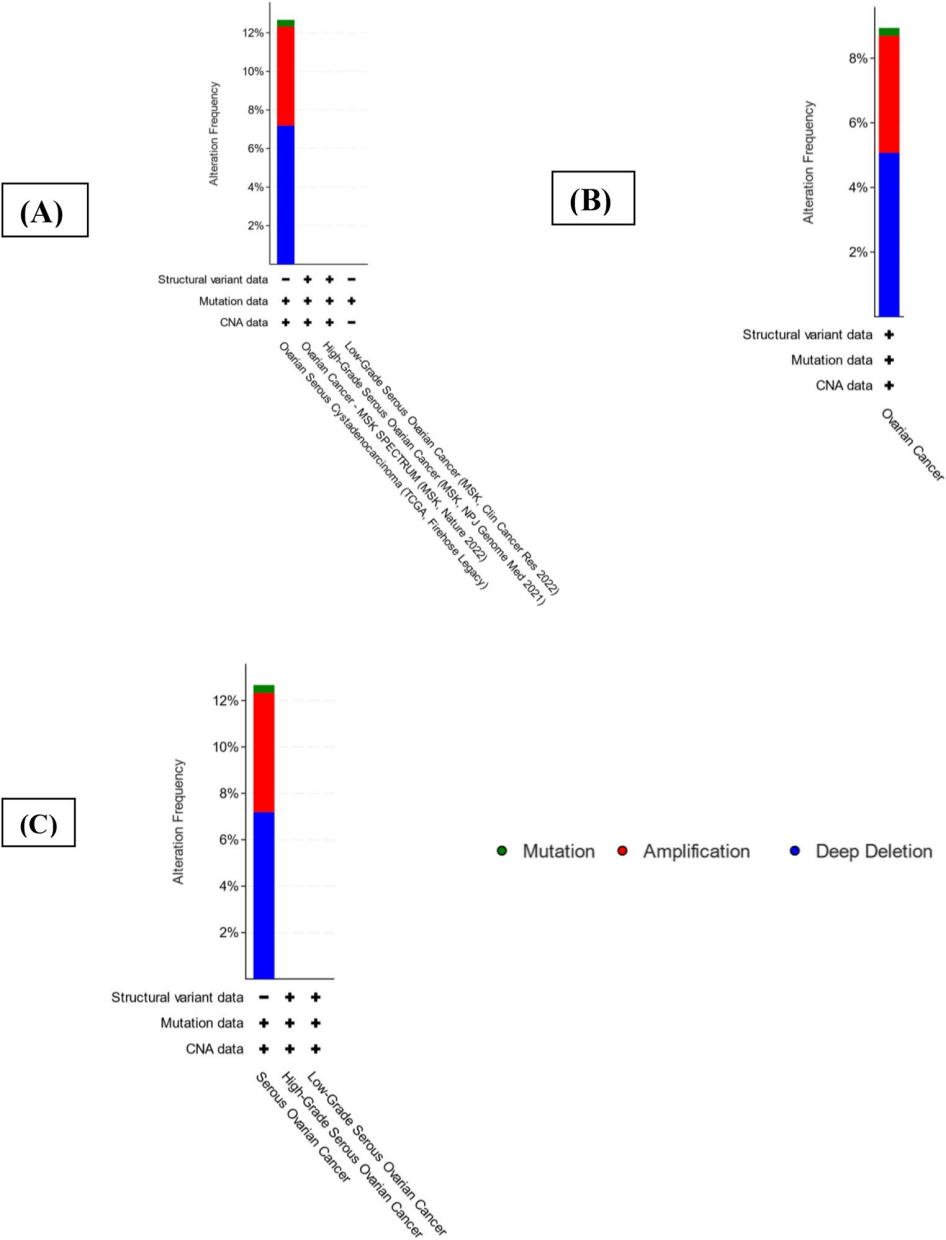
Barplots depicting (**A**) Alteration frequency of all four key genes in different cohorts. (**B**) Alteration frequency of all four key genes in different cancer types (**C**) Alteration frequency all four key genes in detailed sub cancer types

The selected significant genes having p-value less than 0.005 is taken into consideration and (ELAVL2, MAF, ZNF532 and BCL2) were entered to cBioPortal for verifying their variation in genetics throughout 4 different groups incorporating overall 863 samples in 806 patients in 4 studies. All the selected cohorts are from the samples of epithelial ovarian cancer. Figure 3 shows an OncoPrint demonstrating the genetic modification frequency of key DEGS. Further to study the alteration frequency in different cancer types and sub types, bar plots are constructed that shows the alteration frequency in different cohorts showcasing different cancer type; the subtype and the cancer study Fig. 4. The green, red and blue colour demonstrates the mutation amplification and deep deletion respectively in different cohorts. The symbols **+** and – shows the number of profiles that has been profiled or not profiled respectively in different cohorts.

Summary based on all the cancer study from different cohorts shows that gene is altered in 12.67% of 584 cases with mutational frequency of 0.34% (2 cases), amplification frequency of 5.14% (30 cases) and deep deletion frequency of 7.19% (42cases) in all the queried genes of cohort, Ovarian serous cystadenocarcinoma of TCGA firehouse legacy. (Fig. 4A).

Summary based on analysis of different Cancer type depicts the overall alteration frequency of all the four significant genes as depicted in Fig. 4B indicates gene is altered in 8.94% of 828 cases with a mutational frequency of 0.24% (2cases), amplification frequency of 3.62% (30 cases), and deep deletion frequency of 5.07% (42 cases) in case of ovarian cancer. Figure 4B. The symbols **+** and – shows the number of profiles that has been profiled or not profiled respectively in different cohorts.

Considering the details of cancer sub types, we found that gene is altered in 12.67% of 584 cases with mutational frequency of 0.34% (2 cases), amplification frequency of 5.14% (30 cases) and deep deletion of 7.19% (42 cases) in case of serous ovarian cancer. (Fig. 4C). The symbols **+** and – shows the number of profiles that has been profiled or not profiled respectively in different cohorts.

**Fig. 5:**
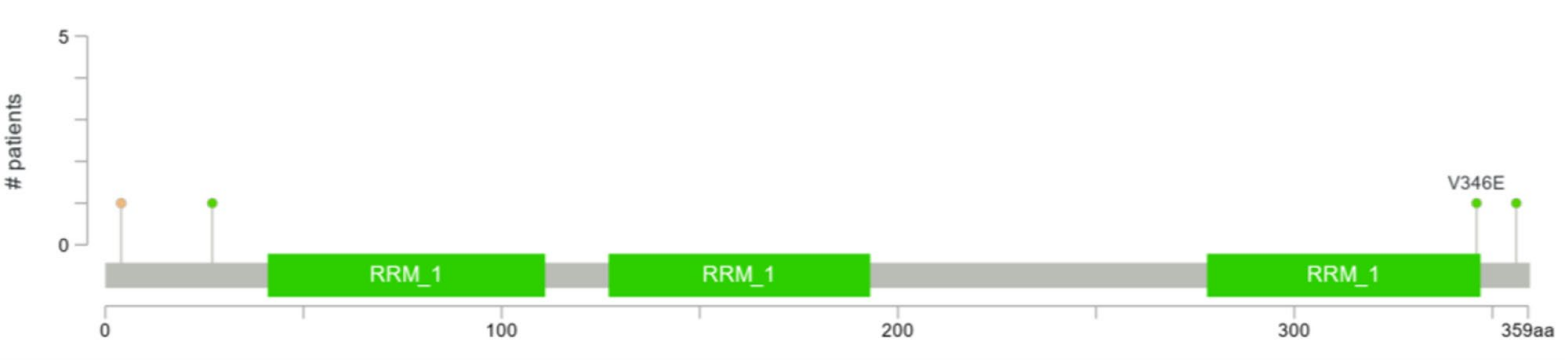
Lollipop plots of somatic mutations in the protein domains of ELAVL2. Below the gray horizontal bar is the amino acid range for the protein domain. Solid green boxes indicate protein domains. Lollipop-like dots on solid vertical lines indicated mutation locations and frequencies

To study the somatic mutations lollipop plots were constructed that indicate the frequency and position of potential mutations in ELAVL2. 0.2% is the percentage of samples with somatic mutations in ELAVL2. No somatic mutations have been found in MAF, ZNF532 and BCL2. (Fig. 5).

### Molecular docking analysis

**Fig. 6:**
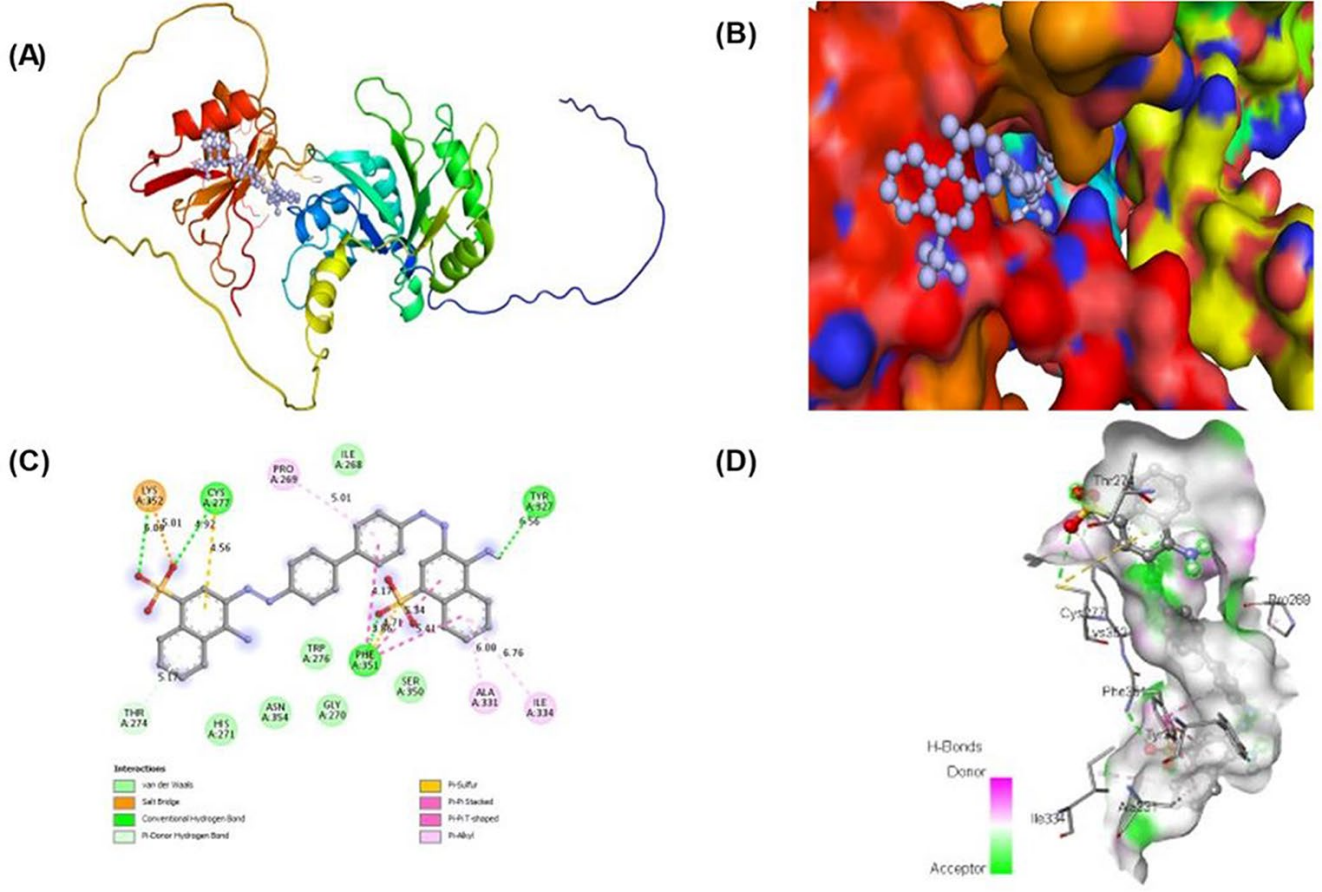
ELAVL2 structure complexed with ZINC03830554. (**A**) A cartoon depiction of the ELAVL2 complex. (**B**) A zoomed surface illustration of ELAVL2 and ZINC03830554 is shown as sticks. (**C**) A close-up of the substrate binding pocket, which shows the critical amino acid residues of ELAVL2 interacting with the inhibitor compound ZINC03830554. (**D**) Surface depiction of ELAVL2’s preserved substrate-binding pocket

Autodock Vina program was used for molecular docking of ELAV-like protein 2. The protein was first retrieved from Uniprot with the accession code Q12926. The ZINC database’s natural compound library was also accessed, and thousands of compounds were evaluated for their capacity to bind to the protein. The compounds with the highest binding energies, namely ZINC03830554, ZINC03830332, ZINC03830328, ZINC03830649, and ZINC03831622, were chosen from Table 2. Subsequently, an in-depth investigation was conducted utilizing PyMOL and discovery studio visualizer to determine the compound exhibiting the most significant residual interaction (Supplementary Material-S1). Based on these results, one compound ZINC03830554 which shown **(**Fig. 6**)** highest interactions towards ELAV-like protein 2 was chosen for further investigation.

ELAVL2 structure contains a long chain of 359 amino acids, having 3 major domains apart from disordered domain. Region (aa 1–33) disordered, domain 39–117 RRM1, domain125-205 RRM2 and domain 276–359 RRM3. The domain containing residues LYS352, CYS277, TYR327, and PHE351is very important because of the presence of potentiality of ligand-interacting motif. It has been observed that the interaction between these interacting binding site residues (LYS352, CYS277, TYR327, and PHE351) has been shown to stop or significantly reduce in the catalytic activity of ELAVL2. The other interacting residues, ASN126, ASN205, LYS352, CYS277, TYR327, and PHE351, SER118, ARG123, THR153, ARG172, SER207, ASN44 and ARG172 belong to the three major domains of ELAVL2 and are also the part of interacting motif and participates in its activity. The ZINC03830554 bound with crucial residues such as LYS352, CYS277, TYR327, and PHE351, these residues formed conventional hydrogen bonds and THR 274 formed Pi-donor hydrogen bond which are very important. Other interactions such as salt bridge, pi-sulfur, pi-pi stacked, pi-pi T-shaped, pi-alkyl and Van der Waals forces was also observed. Distance between bonds was also measured. We found the residual interaction of all the five residues and the findings are reported in Supplementary Material S1. All the residual interaction files can also be found in Supplementary Figures. The compound ‘ZINC03830554’ showed greater binding affinity along with a greater number of interactions towards target protein as compared to other zinc compounds so its is further selected for molecular dynamics simulations.

Also, using Eq. 1, the protein’s inhibitory constant (Ki) value was computed (Table 2). It serves as a measure of the inhibitor’s efficacy; an inhibitor with a low Ki value is one that is effective [32].

**Table 2:** The docking score of different compounds with ELAVL2

S. No.	Compounds	Targeted protein	Affinity (kcal/Mol)	Inhibition constant (nM) KI
1.	ZINC03830554	ELAV-like protein 2	-9.3	1.52407E-07
2.	ZINC03830332		-9.4	1.28737E-07
3.	ZINC03830328		-9.4	1.28737E-07
4.	ZINC03830649		-9.1	2.13602E-07
5.	ZINC03831622		-9.1	2.13602E-07

1 $$KI = {\rm{EXP}}\left( {\left( {{\rm{\Delta G *1000}}} \right){\rm{/}}\left( {{\rm{R*T}}} \right)} \right)$$

Where ΔG = docking energy; *R* = 1.98719 cal K^− 1^ mol^− 1;T 298.15°k^, Ki = inhibition constant (nM).

Apart from various other reasons there are three main reasons for choosing ZINC03830554 as a potential inhibitor for further studies:

- The compound ‘ZINC03830554’ has been shown to reduce cell proliferation and tumor growth. ZINC03830554’ is reported as potential inhibitors of mutant PARP12 receptors [33].
- Apart from this, ZINC03830554’ is among one of the potential inhibitors ranked by Z-mean value that is used as structure-based virtual screening method against Cz protein from T. cruzi [34].
- In addition, a recent study found that ZINC03830554 (also known as Congo red) reduced the proliferation of cell of MLH1 deficient HCT116 in cells from humans with colon cancer and suppressed tumor growth [35].

Lastly, it is noteworthy that ZINC03830554 has exhibited the highest level of interaction with ELAVL2 and showed greater binding affinity along with interactions towards target protein as compared to other zinc compounds. Therefore, on further clinical studies and experimental validation we can assume that ZINC03830554 can also be used to suppress the cell proliferation and tumor growth in ovarian cancer as well. Here, out of 5 compounds, structural representation ZINC03830554 in complexed with ELAVL2 is shown which was selected to perform MD simulation studies **(**Fig. 6**).**

### Physicochemical and ADME/T studies

The top five hits on the shortlist, notably, ZINC03830554, ZINC03830332, ZINC03830328, ZINC03830649, and ZINC03831622 compounds were utilized to determine pharmacologic characteristics such as Molecular Weight, DonorHB, Lipnski violations and AcceptorHB. Four compounds broke one rule by increasing their MW, but this is still permitted. According to the RO3 investigation, the compound’s molecular weight ranged from 422 to 693, with 4–10 H-bond accepting and 0–14 H-bond donor. We discovered that all five compounds followed the rule of three (Ro3) [36]. There are various exceptions to the rule that an orally delivered drug/compound shouldn’t be in violation of more than one rules [37]. SwissAdme is the most extensively utilized tools in rational drug discovery are in-silico absorption, distribution, metabolism, excretion (ADME), and toxicity (T), which provide a good understanding of the drug candidateship. We investigated the ADMET (Ro3) of the top five hits in this context, and the findings are reported in Supplementary Table S2. Overall, the five hits demonstrated adequate drug-related characteristics and skills [38, 39].

### Molecular dynamic simulations

#### ROOT-mean-square deviation

**Fig. 7:**
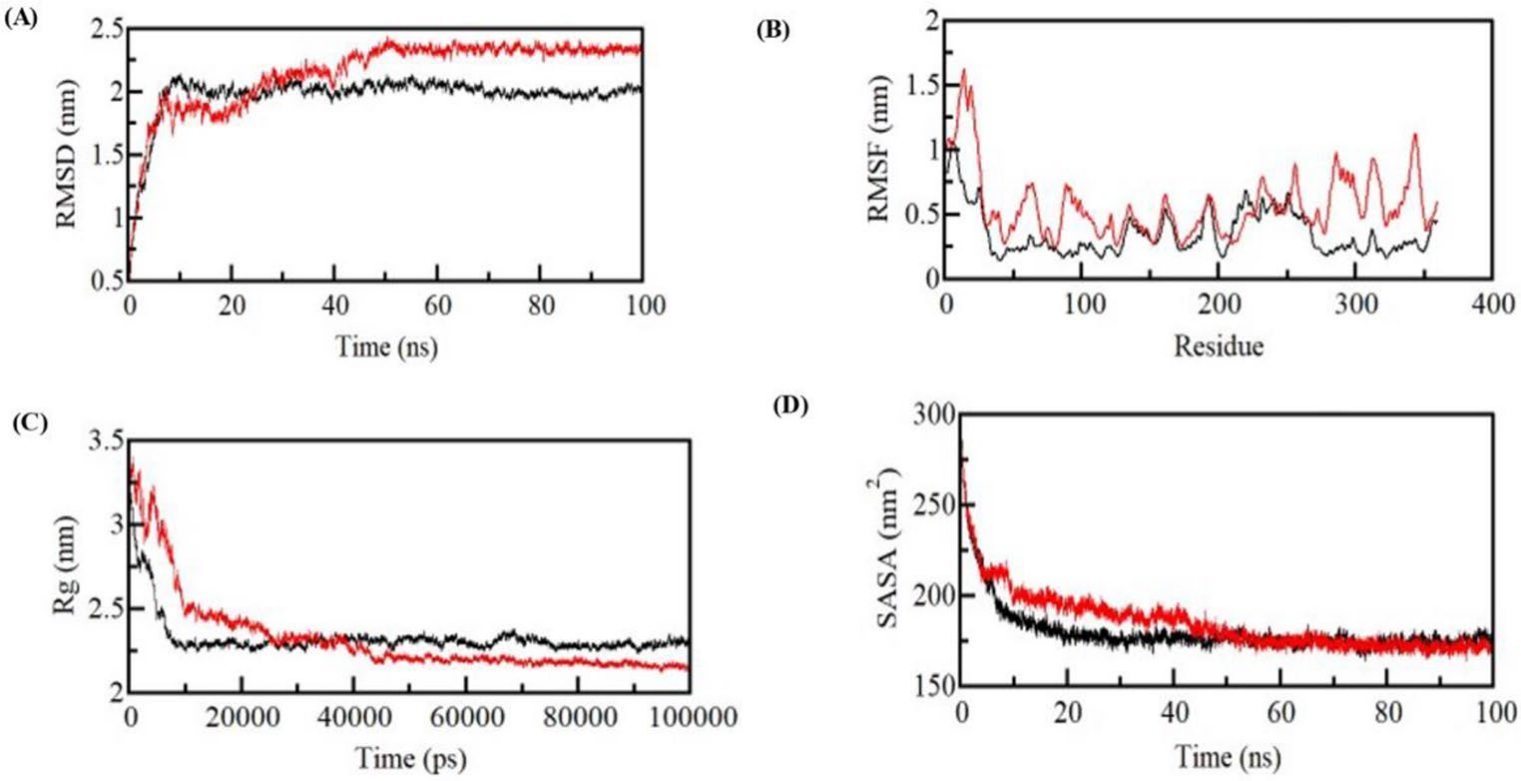
Structural dynamics of ELAVL2 upon ZINC03830554. (**A**) RMSD plot of ELAVL2 in complexed with ZINC03830554. (**B**) RMSF plot of ELAVL2 and its complex with ZINC03830554. Structural compactness and folding of ELAVL2 upon ZINC03830554. (**C**) Rg plot and (**D**) SASA plot of ELAVL2 with ZINC03830554

RMSD is frequently used metric to examine the structural resemblance between two conformations of a molecule, such as a protein or a protein-ligand complex, obtained from molecular dynamics (MD) simulations. In this study, we calculated the RMSD values for both the protein and the protein-ligand complex and compared their values. Our analysis revealed that the average RMSD value for the ELAV-like protein 2 was 0.55 nm, indicating a relatively stable structure during the simulation. Alternatively, the average RMSD value for the ELAV-like protein 2-ZINC03830554 complex was 0.56 nm, which was somewhat higher than that of the ELAV-like protein 2 alone, suggesting that the binding of the ligand may have induced some conformational changes in the protein. However, the deviation of the average RMSD value for the ELAV-like protein 2-ZINC03830554 complex was bit higher than that of the protein alone, indicating that the binding of the ligand may have also retain the protein structure stability. Overall, our results suggest that the ELAV-like protein 2-ZINC03830554 complex was relatively stable during the simulation **(**Fig. 7**)**.

#### ROOT-mean-square fluctuation

RMSF is a measure of the average deviation of each atom in a protein or protein-ligand complex from its average position over the course of a molecular dynamics (MD) simulation. In this case, the average RMSF of the ELAV-like protein 2 was 0.23 nm, indicating that the protein was stable during the simulation. However, the average RMSD of the ELAV-like protein 2-ZINC03830554 complex was 0.26 nm, which is higher than the average RMSF of the protein alone. This implies that the ligand’s adherence to the protein may be causing small fluctuations in the protein structure, potentially indicating some degree of instability in the complex. Overall, the results suggest that while the protein is relatively stable, the addition of the ligand introducing some level of flexibility in the complex although overall protein gets their stability **(**Fig. 7**)**. Further analysis, such as examination of specific regions of the protein and ELAV-like protein 2-ZINC03830554 interactions, may be required to better comprehend the significance of these fluctuations and their potential impact on the structure and function of the complex.

#### Radius of gyration

In the present study, Rg was used as a measure of the compactness of the ELAV-like protein-2 and its complex with the ligand ZINC03830554. The Rg values were calculated from the MD simulation trajectories and the average Rg values were determined for both the protein and the protein-ligand complex. The average Rg of the ELAV-like protein-2 was found to be 2.32 nm, indicating that the protein is compact in its native state. Similarly, the average Rg of the ELAV-like protein-2-ZINC03830554 complex was also found to be 2.32 nm, suggesting that the binding of the ligand did not considerably alter the compactness of the protein. These results indicate that the binding of ZINC03830554 did not induce any major conformational changes in the ELAV-like protein-2, and that the protein remained relatively compact throughout the simulation **(**Fig. 7**)**. The Rg values obtained in this study provide valuable perspective into the structural dynamics of the ELAV-like protein-2 and its interaction with ZINC03830554 and could be useful in the design of new drug candidate for this target.

#### Solvent-accessible surface area

The findings of the SASA analysis suggest a marginal elevation in the mean SASA following the interaction between the ELAV-like protein-2 and the ligand ZINC03830554. The mean solvent-accessible surface area (SASA) of the ELAV-like protein-2 was found to be 180.23, whereas the mean SASA of the ELAV-like protein-2-ZINC03830554 complex was determined to be 185.13. The observed rise in solvent-accessible surface area (SASA) implies that the interaction between the ligand and the protein induces a conformational alteration in the protein’s structure, resulting in the unveiling of supplementary surface area (Fig. 7). The SASA distribution, similar to the Rg values, demonstrates a comparable equilibration pattern in both systems without significantly impacting the overall folding and compactness.

#### Hydrogen bond dynamics

**Fig. 8:**
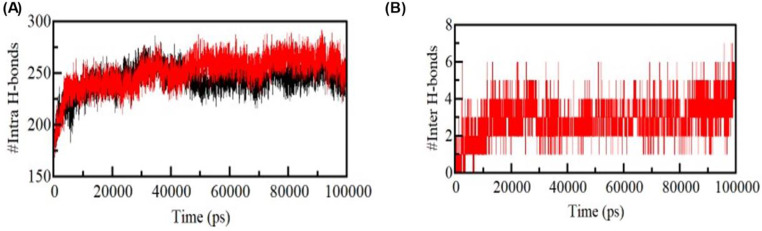
Hydrogen bond analysis. (**A**) Time evolution of intra-molecular H-bonds. (**B**) Figure shows time-evolution of intermolecular hydrogen bonds formed between ELAVL2 upon ZINC03830554

Hydrophobic interactions and intramolecular hydrogen bonds (H-bonds) determine the structural form of a protein. H-bonds inside a protein are crucial to the structure’s overall folding and conformation. Protein structure conformational changes and compactness have long been studied using intramolecular H-bonds. The ELAVL2 protein generated an average of 243 intramolecular hydrogen bonds, while ZINC03830554 interaction generated an average of 251 intramolecular hydrogen bonds **(**Fig. 8**)**. These results suggest that the binding of the ZINC03830554 ligand to the ELAVL2 protein has a slight effect on the formation of intramolecular hydrogen bonds. The increase in the average number of intramolecular hydrogen bonds in the protein-ligand complex may indicate that the binding of the ligand promotes a more stable conformation of the protein. Overall, the MD simulation results suggest that the ELAVL2 protein can form intramolecular hydrogen bonds and that the binding of the ZINC03830554 ligand may affect the formation of these hydrogen bonds. Additional investigations are needed to investigate the functional implications of these results in the context of protein-ligand interactions. Furthermore, the time-dependent assessment of intermolecular H-bonds was investigated to determine the consistency of H-bonding between complexes. (Figure 8 depicts two average numbers of H-bonds, indicating a reasonable consistency for intermolecular H-bonds in the ligand bound position.

#### Secondary structure changes

The computation of secondary structural components of the ELAVL2 protein was conducted in order to monitor alterations in the overall content of its structure over time during the interaction with a ligand.

**Fig. 9:**
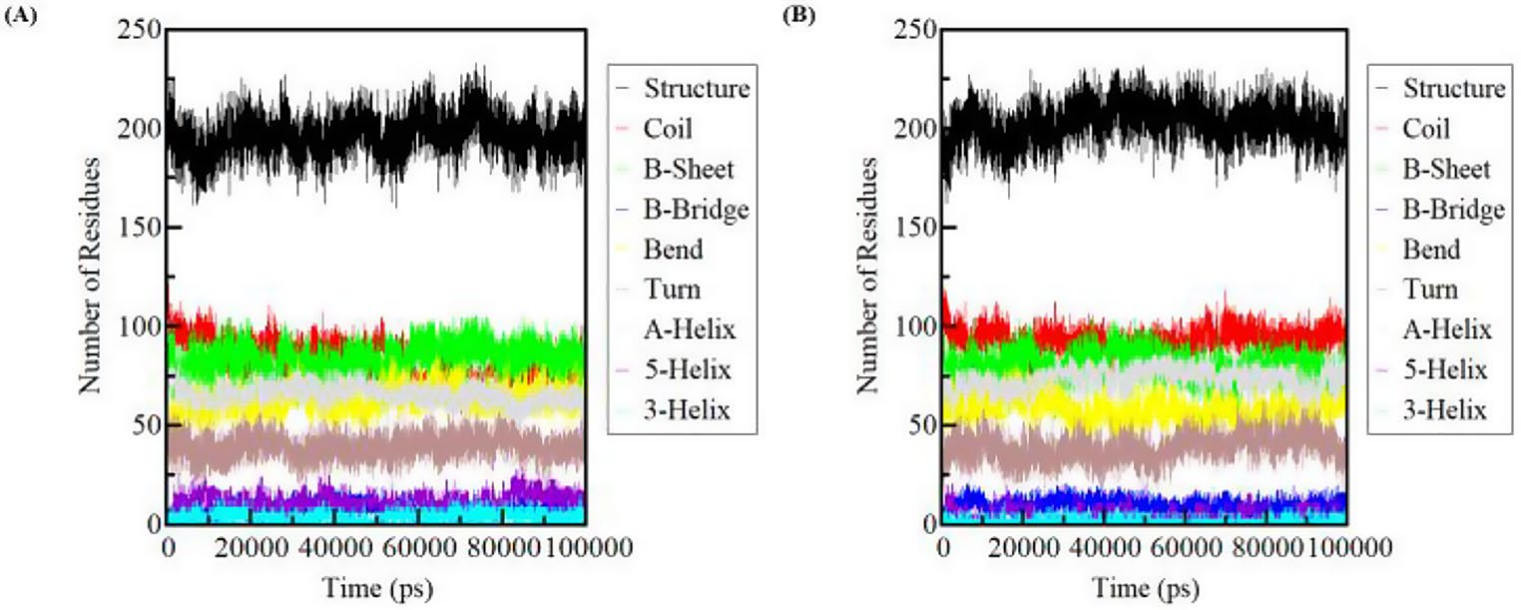
Time dependent secondary structure content. (**A**) ELAVL2 protein. (**B**) ELAVL2 after interaction of ZINC03830554

The molecular dynamics (MD) simulation analysis of the apo ELAVL2 protein and its subsequent interaction with ZINC03830554 shown that there was no substantial alteration in the composition of the secondary structure. However, slight changes were observed in the formation of coils and helices. The secondary structure composition of the apo protein consisted of 0.18% alpha helices, 0.23% beta sheets, and 0.23% coils. After binding the ligand, the secondary structure composition of the protein remained relatively stable with a minor increase in the percentage of coils and a slight increase in the percentage of alpha helices. The new secondary structure composition consisted of 0.21% alpha helices, 0.22% beta sheets, and 0.26% coils Table 3. Overall, the results suggest that the binding of the ligand did not induce significant changes in the secondary structure of the protein, indicating that the ligand may not be affecting the overall conformation of the protein (Fig. 9). However, the observed changes in the formation of coils and helices may indicate localized changes in the protein’s structure, which could potentially impact its function. Further analysis and experimentation are necessary to determine the precise effects of the ligand binding on the protein’s structure and function (See Fig. 9. Table 3).

**Table 3:** Percentage of residues participated in average structure formation

Systems	Structure	Coil	β-sheet	β-bridge	Bend	Turn	A-helix	5-helix	3-helix
*ELAVL2*	0.55	0.23	0.23	0.02	0.18	0.11	0.18	0.03	0.01
*ELAVL2-* ZINC03830554	0.56	0.26	0.22	0.03	0.16	0.11	0.21	0.01	0.00

## Discussion

OC constitutes one of the deadliest cancers, and its survival rates are poor. The current treatments for it include surgery, radiation, and chemotherapy. In perspective behind what distinguishes each cancer subtype apart, there are substantial differences. To ascertain the severity of the ailment, several characteristics might be examined. This could be used to identify effective disease-specific treatment alternatives. The most popular method for locating improperly expressed genes in disease is differential expression analysis. Since the advent of high throughput technologies, there have been numerous studies on OC. By focused therapy, there is a ton of room for the development of novel treatment approaches. To detect DEGs, statistical analysis was carried out on the data sets obtained from GEO. Overall, the nine reported genes [11] are predominantly engaged in various biological processes, and their expression results in an increase in cell number and proliferation, which may eventually result in the development of cancer. By using pathway enrichment analysis, it was discovered that each of these genes was cardinally enriched in several biological processes. Each gene’s specifics have already been covered in our research page. The chosen hub genes work together as a unit and could have a significant impact on OC. All these genes were revealed to be cardinally enriched in several biological processes by pathway enrichment analysis.

We conducted a comprehensive validation of gene expression and observed that the oncogenesis of ovarian cancer is intricately linked to the downregulation of MAF, ZNF532, SCN2A, BCL2, and ELAVL2.Moreover, after the selection of specific genes, a comprehensive survival analysis is conducted to ascertain their prognostic significance in relation to overall survival. Among the five genes under investigation, it was observed that four of them were a statistically significant.

Moreover, in order to determine the mutational status of hub genes in ovarian cancer, we conducted an analysis using the cBioPortal tool. Our findings revealed that the frequencies of alterations were much greater in different genes associated with ovarian cancer. The examination of individual mutations in hub genes has provided insights into the prevalence of alterations in ELAVL2, which exhibited the greatest frequency of 5% in ovarian cancer (Fig. 3). This finding suggests a correlation between genetic alterations and the disruption of normal cellular processes, perhaps leading to the increased expression of these genes. Consequently, this could result in abnormalities within the pathways linked with these genes. In addition, gaining a more comprehensive comprehension of the correlations between somatic mutations and cancer characteristics would be highly advantageous in the development of targeted cancer treatments. Furthermore, developing precision cancer therapy would benefit greatly from a clearer comprehension of relationships among somatic mutations and cancer characteristics. Hence ELAVL2 is taken for further analysis.

In the context of numerous illnesses, including malignancies, RNA-binding proteins (RBPs), a group of naturally occuring proteins that can connect to mRNAs and affect the quantity of protein they create, have received a lot of interest. According to persuasive studies, RBPs may be inappropriately expressed in a variety of cancer cell types and tissues, particularly cells and tissues of OC. RBPs can control carcinogenesis, invasion, metastasis, proliferation, apoptosis, and chemosensitivity, making them attractive therapeutic targets in OC [40]. RNA-binding proteins (RBPs) has the ability to exert control over a diverse range of downstream targets in a comprehensive and multifaceted manner. It is noteworthy that even little disruptions in the expression or activity of RBPs can yield substantial impacts on regulatory networks. In the process of forming ribonucleoprotein (RNP) complexes, RNA-binding proteins (RBPs) have the ability to interact with a diverse range of molecules, such as proteins, messenger RNAs (mRNAs), and non-coding RNAs (ncRNAs) [41]. and then control RNA transcripts’ activities by a variety of posttranscriptional mechanisms, such as RNA splicing, polyadenylation, impacts on localization and stability, and translational modification [41, 42]. Epithelial-mesenchymal transition (EMT), invasion, and metastasis are all impacted by abnormal RBP expression since it can cause genome-wide alterations in the transcriptome and proteome levels. As a result, it is not surprising that RBP expression is frequently altered during the onset and spread of cancer [40].

A type of RNA-binding proteins called Embryonic Lethal Abnormal Vision-Like (ELAVL) proteins were initially discovered to have a crucial role in controlling the growth and functioning of the nervous system. In later research, it was shown that ELAVL proteins had regulatory effects in tissues other than the nervous system (fat cells, hepatocytes, intestinal epithelial cells and vascular smooth muscle cells) [43]. ELAVL2 also called as HuB or Hel-N1 [44]. In addition to being engaged in post-transcriptional and post-translational controls, ELAVL2 is crucial to several other processes. Moreover, it is very important for the brain’s typical cognition and behaviour [45]. In the regulation of spermatogonia development and apoptosis, ELAVL2 plays post-transcriptional activities [46]. High ELAVL2 expression has been linked to 60% of small-cell lung malignancies, according to studies [47]. ELAVL1 (HuR), a homolog of ELAVL2, has recently been found to be significantly expressed in colorectal cancer and to have the ability to control the proliferation and migration of tumor cells [48]. TSPAN4 and ELAVL2 protein expression levels are even independently risk indicators for a poor therapeutic response in ESCC patients [49]. When neural stem cells mature, ELAVL2 encourages cell cycle exit, and its overexpression prevents the multiplication of neuronal stem cells [50]. ELAVL type proteins (ELAVL2) have a role in many pathophysiological processes as well as in the regulation of mRNA. Recent research has revealed a connection between ELAVL2 and menopause and primary ovarian insufficiency (POI) [51]. For Mice lacking ELAVL2, are infertile and have follicle-free ovaries [52]. ELAVL2 encodes an RNA-binding protein that is mostly expressed in neurons, testicles, and ovaries [51, 52]. Further It makes logical to create new small molecules given the prevalence of ELAVL2 proteins and their function in several illnesses. It is worthwhile to consider developing small molecule analogs to encourage the breakdown of ELAVL2 proteins or prevent the translation of their mRNAs based on the mechanism of interaction between small molecule medicines and ELAVL2 proteins. Another strategy to lessen the function of the protein family is to create chemically inert analogs of the ELAVL2 proteins to compete with small molecule therapies or disrupt their synergy [43].

The primary factor in failure of chemotherapy and individuals death with advanced ovarian cancer is resistance to multiple drugs being administered [53]. To increase the rate of survival in patients with advanced OC, drug resistance must be addressed. Many malignant tumours, particularly ovarian cancer, have high expression levels of the RNA-binding protein family known as ELAVL. The ELAVL protein plays a role in drug resistance, growth of tumours, and carcinogenesis. Resistance to Chemotherapy in ovarian cancer is a result of high ELAVL2 expression of protein in tumour cells that encourages tumour growth, invasion, and migration, disturbs the cell cycle, and blocks tumor cell apoptosis brought on by chemotherapeutic medicines through a variety of routes. Notably, even though the ELAVL protein family has a promising future as a therapeutic target, there are still a lot of unanswered concerns. Although interfering with ELAVL proteins appears to be a novel tactic, it must be carefully considered whether doing so may have unintended consequences given that they are crucial for life processes and interact with many RNA molecules. Another approach to lessen the functioning of the protein family is to create chemically inactive analogs of the ELAVL2 proteins to compete with small molecule medications or disrupt their synergy. They play a crucial role in life processes and interact with a lot of RNA molecules, so it’s important to carefully assess if their interference would have additional unintended consequences. Is it preferable to take it by itself or in conjunction with other medications? According to earlier studies, ELAVL1 inhibition made tumors more sensitive to the effects of platinum-based medications like cisplatin and oxaliplatin [54]. Also, a significant amount of research is urgently needed to close the gaps in the development of ELAVL2 inhibitors given the involvement of ELAVL2 in numerous clinical processes and the structural similarity between ELAVL2-4 and ELAVL1.

Several mRNAs are stabilized by ELAVL proteins by binding to the 3′-untranslated region, which promotes the growth of cancers such as ovarian cancer, hepatocellular carcinoma, pancreatic cancer, breast cancer etc. Our strategy looks for compounds with improved pharmacokinetics and higher binding affinities, which may subsequently be examined for prospective pharmaceutical alternatives. These compounds were obtained from chemical libraries, and careful inspection of the docking data revealed, that these chemicals bind properly. These compounds predicted ADMET properties were further examined, and five compounds were ultimately chosen ZINC03830554, ZINC03830332, ZINC03830328, ZINC03830649, and ZINC03831622 to check the stability of protein ligand complex. Overall, our results suggest that the ELAV-like protein 2-ZINC03830554 complex was relatively stable during the simulation.

Our research has identified a number of new substances that have promising therapeutic and prognostic properties, which have not been observed in the context of ovarian cancer (OC) before. Based on evidence, these targets are regularly implicated in the dysregulation of the genetically modified cell cycle and the evasion of immunosurveillance, which are widely recognized as two prominent hallmarks of cancer. Additionally, our investigation unveiled the roles of these genes in the disruption of the cell cycle and immunosurveillance. Consequently, further examination of these genes has the potential to establish their significance in the advancement of personalized cancer therapies. Furthermore, this proposition is a speculation that is substantiated by the network discovered by bioinformatics research. However, comprehensive investigations conducted in controlled laboratory settings and living organisms are still imperative in order to validate the underlying mechanisms.

## Conclusions

In our earlier meta-analysis, 1856 DEGS were discovered, with 741 and 1115 of them being downregulated and upregulated, respectively [11] The downregulation of BCL2, MAF, ZNF532, and ELAVL2 in OC carcinogenesis warrants additional research as potential prognostic markers. The gene with the greatest number of mutations ELAVL2, is selected for therapeutic intervention. After docking and investigations of the ADMET characteristics, the following five compounds were shown to be interesting therapeutic candidates: ZINC03830554, ZINC03830332, ZINC03830328, ZINC03830649, and ZINC03831622. The compound ‘ZINC03830554’ showed greater binding affinity along with interactions towards target protein as compared to other zinc compounds. Overall, our results suggest that the ELAV-like protein 2-ZINC03830554 complex was relatively stable during the simulation. However, experimental validation is further required to validate the above processes which will be continued in third research article.

### Electronic supplementary material

Below is the link to the electronic supplementary material.

Supplementary Material 1

Supplementary Material 2

Supplementary Material 3

## Data Availability

The dataset used in the current study is publicly available at the NCBI GEO dataset for ovarian cancer. The persistent link can be found at: https://www.ncbi.nlm.nih.gov/geo/query/acc.cgi?acc=GSE119055.

## Abbreviations

OC: Ovarian Cancer
GEO: Gene Expression Omnibus
miRNA: Micro RNA
DEMs: Differentially Expressed miRNAs
DEGs: Differentially Expressed Genes
OS: Overall Survival
PFS: Progression Free Survival
