# Supplementary material for: Unravelling driver genes as potential therapeutic targets in ovarian cancer via integrated bioinformatics approach
Source: J Ovarian Res. 2024 Apr 23;17:86. doi: 10.1186/s13048-024-01402-7 (PMC11036584; doi:10.1186/s13048-024-01402-7)
Supplement: Supplementary file 1 — Supplementary Material 1 [file 13048_2024_1402_MOESM1_ESM.docx]

**Supplementary Table S2: Compounds following Lipinski rule of five/three.**

| **S. No.** | **Compound ID** | **Binding**  **Affinity**  **(Kcal/Mol)** | **M W** (Da) | **LogP** | **H bond Donor** | **H bond Acceptor** | **rBonds** | **Lipinski violations** |
| --- | --- | --- | --- | --- | --- | --- | --- | --- |
| 1. | ZINC03830328 | -9.4 | 641.5 | 4.25 | 4 | 5 | 6 | 1 |
| 2. | ZINC03830332 | -9.4 | 606.5 | 1.21 | 3 | 13 | 7 | 2 |
| 3. | ZINC03830554 | -9.3 | 650.6 | 2.08 | 2 | 10 | 7 | 2 |
| 4. | ZINC03830649 | -9.1 | 448.6 | 5.05 | 0 | 4 | 2 | 1 |
| 5. | ZINC03831622 | -9.1 | 646.6 | 2.45 | 8 | 13 | 6 | 3 |
|  |  |  |  |  |  |  |  |  |

**Table:** ADMET properties of the selected compounds

|  | **Absorption** | **Distribution** | **Metabolism** | **Excretion** | **Toxicity** |
| --- | --- | --- | --- | --- | --- |
| **Compound ID** | ***GI*** | ***Water BBB*** | ***CYP2D6*** | ***OCT2*** | ***Hepato*** |
|  | ***Absorption*** | ***Solubility Permeation*** | ***Inh/Subs*** | ***Substrate*** | ***Toxicity*** |
| ZINC03830328 | High | Less Soluble No | No | No | No |
| ZINC03830332  ZINC03830554 | Low  Low | Less Soluble No  Less Soluble No | No  No | No  Yes | No  No |
| ZINC03830649 | High | Less Soluble Yes | No | No | No |
| ZINC03831622 | Low | Less Soluble No | No | No | Yes |
