# Supplementary material for: Unravelling driver genes as potential therapeutic targets in ovarian cancer via integrated bioinformatics approach
Source: J Ovarian Res. 2024 Apr 23;17:86. doi: 10.1186/s13048-024-01402-7 (PMC11036584; doi:10.1186/s13048-024-01402-7)
Supplement: Supplementary file 2 — Supplementary Material 2 [file 13048_2024_1402_MOESM2_ESM.docx]

**
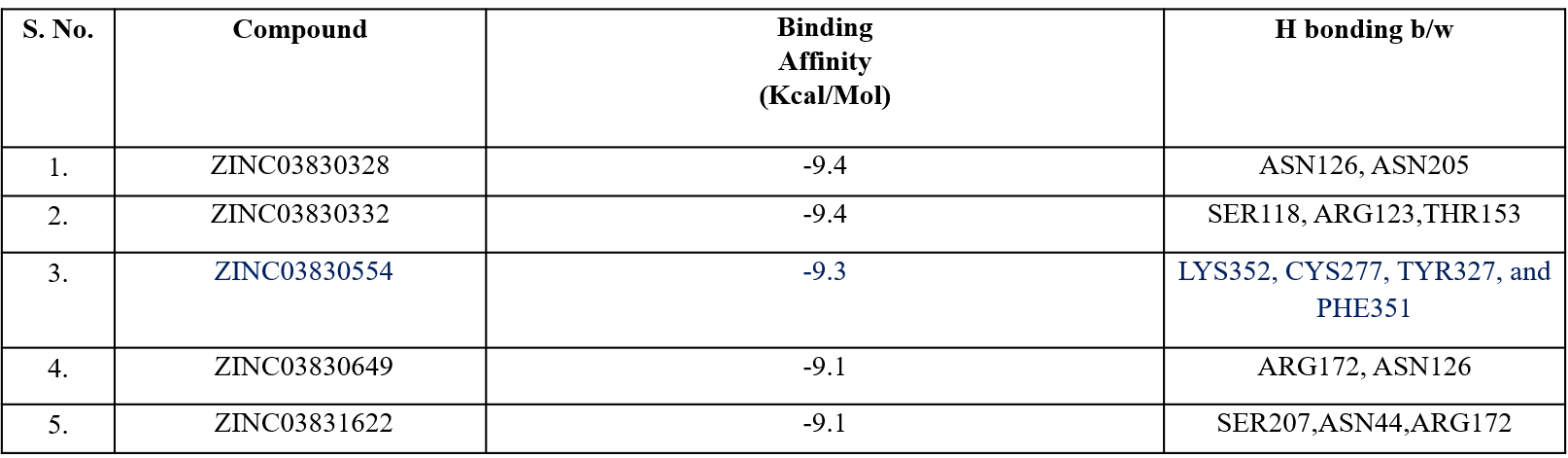
Supplementary Table S1: Compounds interacting with active-site residues of ELAVL2**


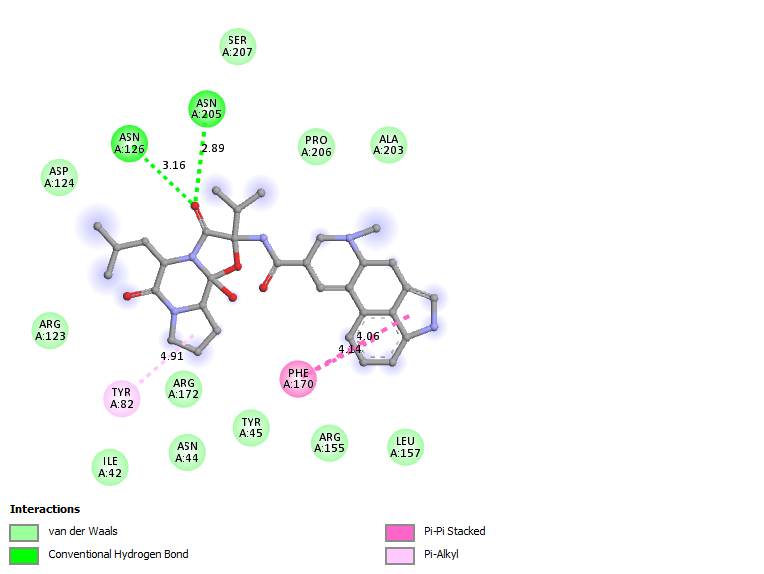


**(1)-ZINC03830328**


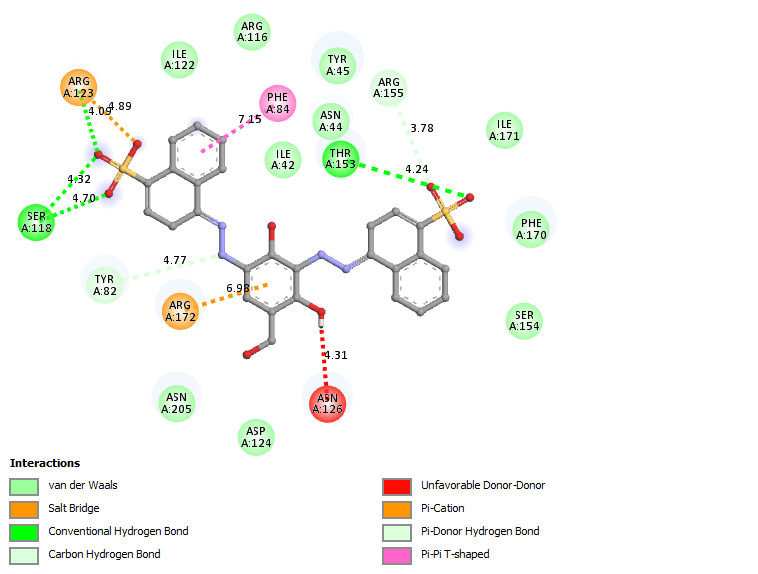


**(2) ZINC03830332**


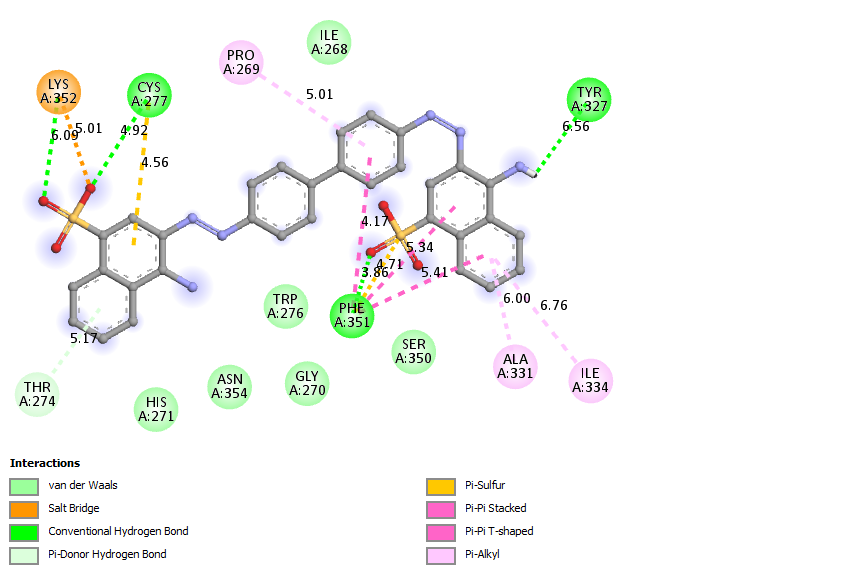


**(3) ZINC03830554**


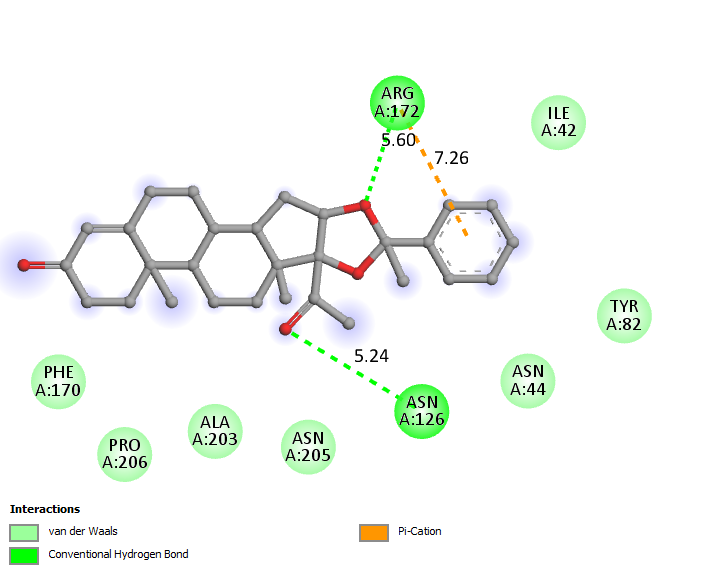


**(4) ZINC03830649**


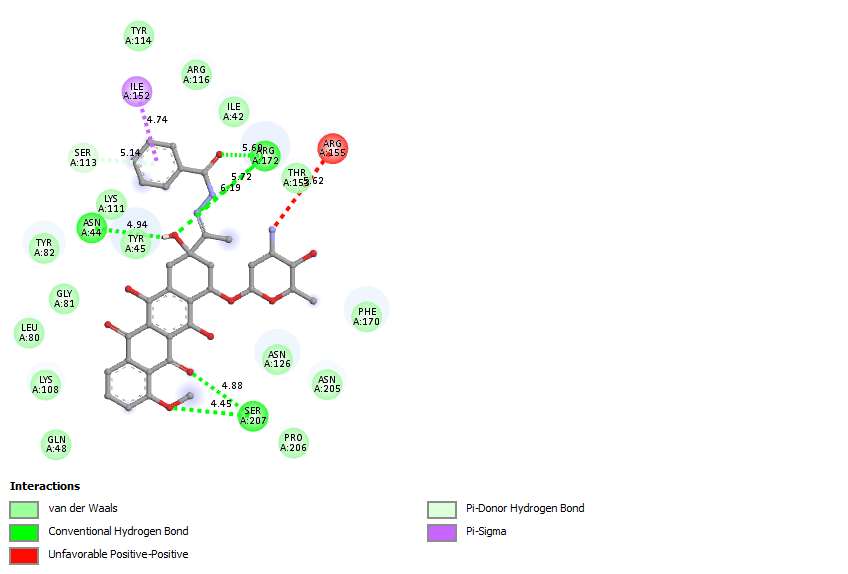


**(5) ZINC03831622**

**Figure**-2D plots of ELAVL2 residues interacting with all the compounds
