# Supplementary material for: Unravelling driver genes as potential therapeutic targets in ovarian cancer via integrated bioinformatics approach
Source: J Ovarian Res. 2024 Apr 23;17:86. doi: 10.1186/s13048-024-01402-7 (PMC11036584; doi:10.1186/s13048-024-01402-7)
Supplement: Supplementary file 3 — Supplementary Material 3 [file 13048_2024_1402_MOESM3_ESM.docx]

**Supplementary Material -3**

Our previous published research article [8] concludes that genes SCN2A, BCL2, MAF, ZNF532, CADM1, ELAVL2, and ESRRG were downregulated in ovarian cancer while genes PRKACB and TAOK1 were upregulated.

Results from GEPIA2 analysis confirmed the downregulation of i.e., SCN2A, ELAVL2, ZNF532, MAF and BCL2.

However, after extensive literature survey we further validated the expression, which concluded the downregulation of CADM1[1-3] and ESRRG[4] as per our previous reports. Further as per previous reports the upregulation of TAOK1 and PRKACB is being reported in various research article[5-7].

However extensive in-vitro and in-vivo research is required to validate the expression levels of all the 9 genes which will be continued in third research article .

**References:**

1. Si, X., et al., *CADM1 inhibits ovarian cancer cell proliferation and migration by potentially regulating the PI3K/Akt/mTOR pathway.* Biomedicine & Pharmacotherapy, 2020. **123**: p. 109717.
2. Li, C., et al., *miR-486 promotes the invasion and cell cycle progression of ovarian cancer cells by targeting CADM1.* Analytical Cellular Pathology, 2021. **2021**.
3. Li, H., J. Gao, and S. Zhang, *Functional and clinical characteristics of cell adhesion molecule CADM1 in cancer.* Frontiers in Cell and Developmental Biology, 2021. **9**: p. 714298.
4. Schueler-Toprak, S., et al., *Expression of estrogen-related receptors in ovarian cancer and impact on survival.* Journal of Cancer Research and Clinical Oncology, 2021. **147**(9): p. 2555-2567.
5. Machino, H., et al., *The metabolic stress-activated checkpoint LKB1-MARK3 axis acts as a tumor suppressor in high-grade serous ovarian carcinoma.* Communications Biology, 2022. **5**(1): p. 39.
6. Dou, Y.-D., et al., *Integrated microRNA and mRNA signatures in peripheral blood lymphocytes of familial epithelial ovarian cancer.* Biochemical and Biophysical Research Communications, 2018. **496**(1): p. 191-198.
7. Li, G., et al., *Prognostic value of the tumor‐specific ceRNA network in epithelial ovarian cancer.* Journal of cellular physiology, 2019. **234**(12): p. 22071-22081.
8. Beg, A., Parveen, R., Fouad, H., Yahia, M. E., & Hassanein, A. S. (2023). Identification of Driver Genes and miRNAs in Ovarian Cancer through an Integrated In-Silico Approach. *Biology*, *12*(2), 192. https://doi.org/10.3390/biology12020192
